# Hierarchical Delivery of Anti-Inflammatory Compound and Stem Cells for Chronic Wounds

**DOI:** 10.3390/pharmaceutics17121549

**Published:** 2025-11-30

**Authors:** Petras Winkler, Ryan Zhang, Yong Mao

**Affiliations:** Department of Chemistry and Chemical Biology, Rutgers University, 145 Bevier Rd, Piscataway, NJ 08854, USA; pjw103@scarletmail.rutgers.edu (P.W.); rz436@rwjms.rutgers.edu (R.Z.)

**Keywords:** GelMA microparticles, hMSCs, curcumin, chronic wounds, THP-1, anti-inflammation

## Abstract

**Background:** Chronic wounds, especially in diabetic patients, pose a significant clinical challenge due to current treatment limitations and an increasingly affected population. A major issue is the stalled inflammatory phase, which prevents proper healing. This study developed a novel co-delivery system to address the deficiency of growth factors and persistent inflammation in chronic wounds. **Methods:** Gelatin nanoparticles (NPs) were synthesized to carry curcumin, an anti-inflammatory compound. These curcumin-loaded NPs (NP/Curc) were then incorporated into gelatin methacrylate (GelMA) hydrogels to form a hierarchical delivery construct, nanoparticles encased within a hydrogel. These hydrogels were then cryogenically milled into microparticles (MPs) to carry human mesenchymal stem cells (hMSCs). The viability and growth of hMSCs on the surface of curcumin-loaded MPs were evaluated. The release of curcumin from various MP configurations was analyzed. The anti-inflammatory effects of the MPs were assessed by measuring pro-inflammatory cytokine expression in human monocyte THP-1 cells. **Results:** Curcumin directly loaded into hydrogels showed a rapid burst release within three days. In contrast, NP/Curc had a more sustained release profile. Curcumin incorporation did not adversely affect the cell-carrier functions of MPs. Conditioned media from hMSCs cultured on the plain MPs demonstrated anti-inflammatory effects in THP-1 cells. Low doses of curcumin released from the MPs also showed anti-inflammatory activity. The combination of hMSCs and curcumin exhibited an additive effect in reducing IL-8 expression by 4× in THP-1 cells. **Conclusions:** This study demonstrates the feasibility of co-delivering cells and curcumin without compromising the cell viability of hMSCs. Using gelatin nanoparticles as a carrier prolongs curcumin release, offering a sustained therapeutic effect. This strategy of hierarchical delivery of curcumin and co-delivery of cells represents a promising approach for treating chronic wounds by simultaneously providing growth factors and reducing inflammation.

## 1. Introduction

Chronic wounds are a significant issue affecting up to 2.5% of the US population as of 2022, a figure which has been steadily increasing over time [1]. Chronic wounds remain in the inflammatory phase of wound healing due to several factors, such as venous insufficiency, diabetes, aging, biofilm formation, deficiency of growth factors, and excessive degradation of the extracellular matrix [2,3,4]. When healing stalls, it leaves persistent, infection-prone open wounds. This vicious cycle significantly worsens the wounds, severely diminishing the patient’s quality of life and causing alarmingly high rates of amputations [5] and sepsis [6].

To overcome the deficiency of growth factors in the wound environment, stem cells have been considered as a remedy. Stem cells are a part of the body’s natural healing process, as they release the relevant growth factors responsible for stimulating epithelial proliferation and angiogenesis [7,8,9,10]. Transplantation of stem cells into the wound environment theoretically will enhance wound healing. However, without the proper surface to attach to, cell viability greatly decreases when transplanting cells to the wound site [11]. To circumvent this issue, cells have been encapsulated into hydrogels (HG) made of natural or synthetic materials, such as gelatin, alginate, chitosan, polyethylene glycol (PEG), and poly(2-hydroxyethyl methacrylate) (PHEMA) [12]. The encapsulated stem cells were then applied to the wound environment [13]. Of special interest are HGs made of gelatin methacrylate (GelMA). GelMA is a modified derivative of collagen that maintains the excellent biocompatibility of the original molecule [14]. While cells encapsulated in HG remain alive, these cells are often not metabolically active due to limited access to nutrients and the decreased rate of diffusion within the HG. Only cells located on or near the surface of the HG maintain their robust metabolic activity, which may correlate with the active production of growth factors [15]. To maximize the functionality of stem cells, a hydrogel system with maximized surface areas would be beneficial as an alternative cell carrier. GelMA microparticles, such as GelMA microspheres (GelMA MS), have been explored as cell carriers [16]. Our previous studies demonstrated that the stem cells cultured on the spherical surface hydrogel produced significantly more factors promoting wound healing than encapsulated cells [9]. Since spheric surfaces have the least surface/volume ratio among all forms of particles, irregularly shaped microparticles formed by cryogenic milling of a GelMA hydrogel may provide even more surfaces for stem cell attachment and growth [17]. Combined with the drug delivery ability of hydrogel, GelMA MPs have the potential to deliver stem cells and wound healing drugs simultaneously.

Curcumin is a botanical agent derived from the *Curcuma longa* species and has shown promising potency as an anti-inflammatory compound [18]. Its mechanism of action involves binding receptors to modulate the NF-κB signaling pathway and regulating reactive oxygen species (ROS) levels in the cell, which leads to lower expression of pro-inflammatory genes such as TNF-α, IL-8, and IL-6 [19,20,21,22,23]. Curcumin’s structure is pH-dependent, existing in diketone or enol forms. Studies investigating how these structural changes affect its efficacy have shown that the major determinants of its anti-inflammatory activity are the presence of aryl groups and the arrangement of functional groups on those rings [24,25]. Therefore, both diketone and enol forms have comparable anti-inflammatory activities. On the other hand, the diketone moiety appears to have little impact [26]. This suggests that curcumin’s antioxidant properties—often associated with the diketone structure—are not the primary contributor to its anti-inflammatory effects.

Curcumin’s efficacy is severely limited by its low solubility in water (approximately 7.4 µg/mL after heating) [27]. It also degrades quickly in an aqueous solution at alkaline pH [28,29,30]. Furthermore, the concentration of curcumin needs to be controlled due to its cytotoxicity at a higher concentration (>25μM) [31]. Therefore, to increase the accessibility of curcumin at a non-cytotoxic dose to cells, a delivery system is required. Pham et al. used a chitosan-P123 hydrogel to deliver curcumin [32]. In a full-thickness wound rat model, they demonstrated that, by day 8, their system had a statistically higher rate of wound closure compared to controls. However, the majority of loaded curcumin was released within the first two hours in vitro in this study. Interestingly, when the hydrogel solution was supplemented with gelatin, the rate of wound closure increased, and the burst release was reduced. This observation suggested that the interaction between gelatin and curcumin prevented the burst release. Similarly, a study reported by Mobaraki et al. synthesized a hydrogel scaffold composed of collagen and sodium alginate. This scaffold showed excellent biocompatibility and was able to mitigate the cytotoxic effects of curcumin, while also showing significantly better wound healing compared to controls after 14 days in a rat excision wound model [33]. However, about 95% of the loaded curcumin was released from the scaffold within 24 h during the release study. To remedy this burst release, nanoparticles have been explored as a carrier for curcumin. Synthetic nanoparticles with hydrophobic cores have been studied for the delivery of curcumin [34].

Gelatin nanoparticles (Gel NPs) have been proven to be an effective nano-carrier for small molecules or proteins/peptides [35]. Yu et al. showed that loading curcumin into poly-γ-glutamic acid nanoparticles significantly reduced the release of curcumin in vitro by about 50% at 12 h. More interestingly, a hierarchical construct was developed in this study by incorporating curcumin-containing nanoparticles into a genipin-crosslinked gelatin hydrogel. This design successfully achieved a further delay in curcumin release. [36].

Due to the multifactorial complexity of chronic wounds, therapies that address multiple deficiencies simultaneously are promising. Supplementing wounds with growth factors via stem cells and controlling inflammatory responses with curcumin (by targeting the NF-κB signaling pathway) should, in principle, provide at least additive beneficial effects on wound healing. Given the known interactions between stem cells and curcumin, combining these two treatments may even yield a synergistic effect [37].

**Figure 1:**
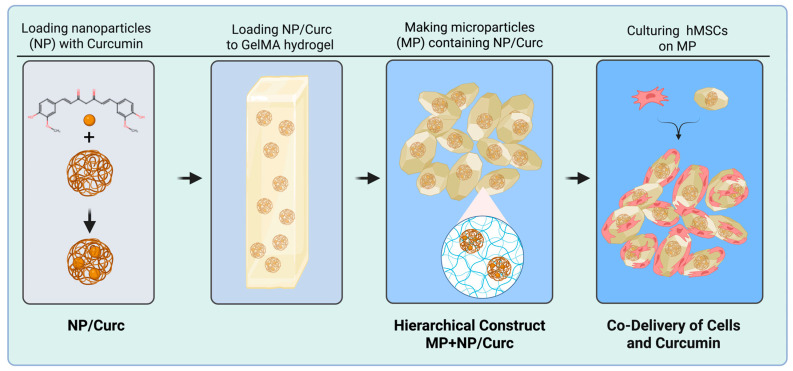
A schematic illustration of the hierarchical co-delivery system.

This study aims to develop a hierarchical scaffold designed for the co-delivery of curcumin and stem cells to the site of a chronic wound. Our goal is to achieve both sustained curcumin release and simultaneous stem cell delivery. To construct this system, we first created gelatin nanoparticles loaded with curcumin. These nanoparticles were then embedded within a hydrogel microparticle matrix, forming the desired hierarchical structure. Stem cells were seeded onto the scaffold to enable the combined therapeutic benefits of both the cells and the gradually released curcumin (Figure 1).

Curcumin can be incorporated into GelMA MP directly during hydrogel fabrication. This direct loading of curcumin is compared with nanoparticle-mediated loading. We showed that loading of curcumin in a hydrogel, at a concentration that otherwise would be cytotoxic, did not detrimentally affect cells cultured on the surface of MPs, which suggested that the release of curcumin was at a level compatible with cells. Loading curcumin into nanoparticles before incorporating them into the microparticle prevented a burst release and created a sustained release profile. As expected, stem cells on GelMA MPs secreted anti-inflammatory factors and reduced the expression of pro-inflammatory markers such as TNF-α, IL-8, and IL-6 in human monocytes, THP-1, in response to LPS stimulation. Curcumin released from MPs showed a similar anti-inflammatory effect on THP-1 cells. The co-delivery of stem cells and curcumin additively reduced the expression of IL-8 in THP-1 cells, even though such an effect was not observed on TNF-α or IL-6. While additional optimization is necessary, this study provides proof-of-concept for the first hierarchical gelatin nanoparticle-GelMA microparticle system designed for the co-delivery of curcumin and hMSCs.

## 2. Materials and Methods

### 2.1. Synthesis of Gelatin Nanoparticles

Gelatin Nanoparticles were synthesized using a modified double desolvation method [38,39]. Briefly, a 5% solution of gelatin was mixed with an equal volume of acetone to precipitate high molecular weight (HMW) gelatin. The HMW gelatin was washed with cold deionized water, redissolved in the original volume, and the pH of this solution was adjusted to 2.5 using 1 M HCl. In a 40 °C water bath, acetone was added dropwise at a rate of 180 mL/h in a 3:1 ratio of acetone to 5% gelatin solution. After, 0.28 μL of 25% glutaraldehyde solution was added for every milligram of starting gelatin mass, in solution to crosslink the nanoparticles for 1 h at 40 °C. The sample was further incubated overnight. To collect the nanoparticles, the solution was centrifuged at 15,000× *g*. The pellets were consolidated and resuspended in 2 mL of deionized water. The pellets were ultrasonicated to disperse them in solution, frozen, and then lyophilized and stored at 4 °C for future use. Particle size was measured using dynamic light scattering using a Zetasizer Nano (Malvern Instruments, Worcestershire, UK).

### 2.2. Loading Nanoparticles with Curcumin

To load curcumin to nanoparticles, 10 mg of dried nanoparticles was weighed out, sterilized, and transferred to a microcentrifuge tube at 25 °C. A 50 µg/mL curcumin solution was prepared by diluting a curcumin stock solution (10 mg/mL in DMSO) in deionized water. 2 mL of this solution was immediately added to the nanoparticles before visible precipitation formed. The mixture was ultrasonicated for 1 min at 50% amplitude on ice (Branson SFX150 Ultrasonicator, Marshall Scientific, Danbury, CT, USA). The microcentrifuge tube was covered in foil and placed on a rotator for 1 h to allow the curcumin to absorb into the nanoparticles. After complete absorption, the sample was then ultrasonicated and used for microparticle synthesis.

### 2.3. Synthesizing Curcumin MPs

GelMA was synthesized by methacrylation of porcine gelatin with methacrylic anhydride as reported previously. The 100% methacrylation of GelMA was determined and confirmed by NMR and ATR-FTIR analyses [40]. Lithium phenyl-2,4,6-trimethylbenzoylphosphinate (LAP) (Sigma-Aldrich, Burlington, MA, USA) was prepared freshly and kept in foil wrap to prevent exposure to light.

The following GelMA solutions were prepared and warmed to 40 °C:

- MP: 10% GelMA *w*/*v*, 0.05% LAP *w*/*v* (freshly prepared and kept in dark)
- MP + Curc: 10% GelMA *w*/*v*, 0.05% LAP *w*/*v*, 100 µM curcumin
- MP + NP/Curc: 10% GelMA *w*/*v*, 0.05% LAP *w*/*v*, 100 µM curcumin, 3.84 mg/mL gelatin NPs)

1.2 mL of the solution was pipetted onto a PDMS-coated 6-well plate. The solutions were solidified using 405 nm UV light for 3 min at a distance of 13 cm. The semi-solidified disks were then flipped and cured for another 3 min. The hydrogels were then frozen at −80 °C overnight. They were then micronized using a mortar and pestle until the average size was approximately 100 μm. Particle size was monitored intermittently during the milling process to ensure that the particles were the appropriate size. The particles were then collected in an Eppendorf tube and lyophilized for 2 days. The dry particles were then stored at 4 °C until use.

MP size was determined by measuring the longest distance between two points on a microparticle using ECHO microscope image capture and analysis (ECHO Revolve, San Diego, CA, USA).

### 2.4. Release and Quantification of Curcumin from MPs

3 mg samples of microparticles (containing 1.1 µg curcumin) were weighed out and sterilized under Sylvania G36T5/SP Germicidal UV-C 39W Fluorescent Lamp for 30 min in a biosafety cabinet. Sterilized MPs were transferred to 1.5 mL microcentrifuge tubes. 0.4 mL of PBS (pH 7.4) was added to each sample and mixed thoroughly to ensure the particles were separated. The samples were placed on a rotator and covered with foil while rehydrating for 1 h. After rehydration, the samples were centrifuged at 12,300× *g* for 5 min, and the rehydration media were collected and labeled as the “Day 0” sample. Samples were then resuspended in 0.4 mL of PBS (pH 7.4) and incubated at 37 °C in the dark and agitated three times per day. PBS was completely changed between each sample collection by centrifuging particles at 12,300× *g* for 5 min to create a pellet, thereby avoiding the withdrawal of MPs. Afterwards, the pellet was resuspended in 0.4 mL of fresh PBS, and incubation was continued. Conditioned PBS was collected at t = 1, 2, 3, and 6 days.

The concentration of curcumin was quantified by measuring the absorbance of samples at 420 nm [29]. A 10 mg/mL solution of curcumin was prepared in DMSO and diluted in PBS (pH 7.4) to 10 μg/mL. Serial dilutions in PBS were used to form a linear standard curve with a range of 10 μg/mL to 0.31 μg/mL. 100 μL of PBS containing released curcumin from each sample was added to a 96-well plate, and absorbance at 420 nm was measured using a TECAN Spark (Spark^®^, TECAN, Mendrisio, Switzerland).

### 2.5. Culturing Human Mesenchymal Stem Cells on GelMA Microparticles

Human bone marrow mesenchymal stem cells (hMSCs) were purchased from Lonza Bioscience (Cat# PT-2501, Walkersville, MD, USA). Passages 4–5 of hMSCs were used for this study. Cells were cultured in MEM-alpha medium (HyClone, Logan, UT, USA) supplemented with 10% fetal bovine serum (CPS Serum, Kansas City, MO) and 25 μg/mL of gentamicin (Sigma-Aldrich, Burlington, MA, USA) in tissue culture-treated polystyrene plates. The plates were incubated at 37 °C with 5% CO_2_ and 95% humidity. When the cell density reached about 80% confluence, cells were trypsinized, counted, and seeded as described previously [17]. Briefly, 3 mg/sample of microparticles (MPs) with or without curcumin were weighed out and sterilized under UV for 30 min. 0.5 mL cell culture media containing 10% FBS was added to each MP sample and mixed by rotating for 1 h to rehydrate MPs while neutralizing any residual aldehyde. The supernatant was discarded after centrifugation at 12,300× *g* for 5 min. 100 µL of fresh media containing 7 × 10^4^ hMSCs (2.33 × 10^4^ cells/mg MPs) was then added to each sample. All samples were placed in the incubator for 30 min to allow cells to attach to the MPs. The samples were then transferred to a 48-well cell-repellant plate (Greiner Bio-One, Kremsmünster, Austria). 0.3 mL/well of cell culture media was then added and incubated in the tissue culture incubator. 0.35 mL of conditioned medium was collected from each well at a predetermined time point and replaced with 0.35 mL of fresh medium.

### 2.6. Calcein AM Staining

After six days of culturing, assay media was replaced with 0.1 μM Calcein AM (Corning Inc., Corning, NY, USA), and incubated in the dark for 30 min at 37 °C. After incubation, the staining media were removed and replaced with assay media while imaging using an ECHO fluorescence microscope.

### 2.7. DNA Quantification of hMSCs Cultured on MPs

0.3 mL of Lysis buffer containing 0.2 mg/mL proteinase K (Sigma-Aldrich, St. Louis, MO, USA) and 0.5% SDS w/v in Tris-EDTA (TE) buffer was added to each sample. The samples were incubated for 1.5 h at 55 °C using a warm bath. After, 0.2 mL of deionized water and 0.5 mL of chloroform phenol isoamyl alcohol (Invitrogen, Carlsbad, CA, USA) were added to all samples. The samples were incubated for 1 h at 25 °C, and then centrifuged at 9960× *g* for 10 min to separate the aqueous, organic, and protein layers. After removing the aqueous layer, 1 mL of ethanol was added to each sample and placed at −20 °C overnight. The samples were then spun down at 9960× *g* for 10 min to precipitate DNA. The supernatant was removed, and the samples were resuspended in 400 μL of TE buffer. The DNA was quantified using AccuClear^®^ Ultra High Sensitivity dsDNA Quantitation Kit (Biotium, Fremont, CA, USA) following the manufacturer’s instructions.

### 2.8. Differentiation and Activation of Human Monocyte Cell Line THP-1

Human monocyte cell line THP-1 was purchased from American Type Culture Collection (ATCC, Manassas, VA, USA). Cells were cultured in suspension with RPMI 1640 (Corning Life Sciences, Kennett Square, PA, USA) supplemented with 10% fetal bovine serum and 25 μg/mL of gentamicin. THP-1 cells were seeded at 2.5 × 10^5^/well in the wells of a tissue culture-treated 48-well plate that contained cell culture medium with phorbol 12-myristate 13-acetate (PMA, Sigma-Aldrich) at 100 nM. After incubating in the tissue culture incubator for three days, THP-1 cells were differentiated and adhered to the surface of the wells. To induce the inflammatory response of differentiated THP-1 cells, lipopolysaccharide (LPS, Sigma-Aldrich) was added to designated wells at 100 ng/mL. After incubation for 24 h, the cells (with and without induction) were lysed using RNA lysis buffer for RNA isolation.

### 2.9. Quantification of Relative Expression of Inflammatory Markers by Quantitative PCR (qPCR)

The relative expression of inflammation markers in human monocyte THP-1 cells was quantified by qPCR as previously described [41]. Briefly, differentiated THP-1 cells were induced with 100 ng/mL of lipopolysaccharide (LPS) in the presence or absence of conditioned medium collected from hMSCs cultured on various microparticles. After 24 h, THP-1 cells were washed with PBS and lysed in 0.2 mL/sample of RNA lysis buffer (Promega, Durham, NC, USA). Total RNA from these lysates was purified using the SV 96 Total RNA Isolation System (Promega, Madison, WI, USA). RNA concentration and purity were measured using the TECAN Spark Nano plate (TECAN, Morrisville, NC, USA). cDNA preparation and qPCR were performed as described [42]. The QuantiTect primer sets used for qPCR were purchased from Qiagen (Germantown, MD, USA) (Table 1). 

Each sample was run in duplicate. After the run was completed, a second derivative analysis was performed using the raw data to determine the mean Cp (Crossing point-PCR-cycle) for each sample. For each gene expression, expression of GAPDH served as an internal control. Relative mRNA expression was determined by Pfaffl analysis (2^ΔCp target^/2^ΔCp reference^), in which ΔCp = mean Cp of sample—mean Cp of the THP-1 cells without treatment (Control). 

### 2.10. Statistical Analysis

All experiments have at least biological repeats (*n* ≥ 3). Statistical analyses were performed using GraphPad Prism version 10.6.1 (accessed on 10 November 2025, GraphPad software) (La Jolla, CA, USA, www.graphpad.com). Comparisons between multiple groups were performed using one-way ANOVA with Tukey’s honest significant difference multiple comparisons test. Differences were considered significant at *p* < 0.05

## 3. Results

### 3.1. Synthesis of NPs and MP Scaffold

**Figure 2:**
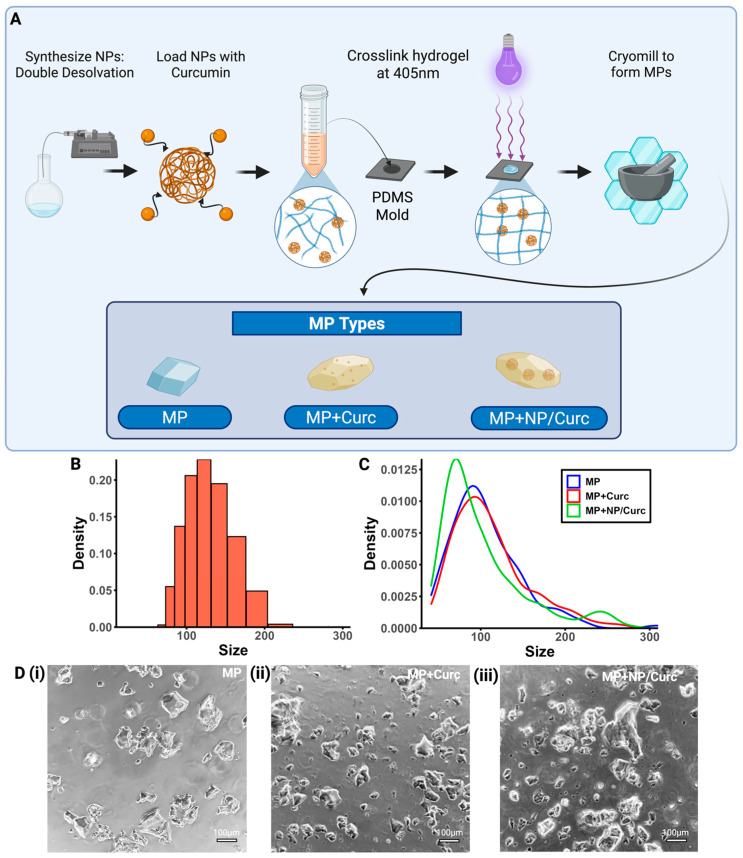
Characterization of MP Scaffolds. (**A**) Schematic overview of the fabrication process. (**B**) Size distribution of gelatin NPs. (**C**) Size distribution of each type of MP. (**D**) Representative images of each MP configuration (scale bar = 100 μm) (**i**) MPs, (**ii**) MPs + Curc100, (**iii**) MPs + NP/Curc100.

Nanoparticles were synthesized using a double desolvation method to act as carriers for curcumin within the hydrogel (Figure 2A). The average hydrodynamic diameter was 118.7 nm, and the PDI of 0.03 indicates that the particles were monodispersed (Figure 2B). NPs synthesized using this method were demonstrated to have a zeta-potential of 24.5 ± 0.8 mV [38,39].

Free curcumin or NP/Curc were then added to the GelMA solution to make various GelMA hydrogels. These hydrogels were cryogenically milled into microparticles (Figure 2A,D). The compositions of each type of MP are below:

- MP (blank): Microparticles containing no curcumin or nanoparticle carriers.
- MP + Curc (free curcumin): Microparticles created from a 10% GelMA *w*/*v* and 100 μM curcumin solution.
- MP + NP/Curc (nanoparticle-loaded curcumin): Microparticles created from a 10% GelMA *w*/*v*, 100 μM curcumin, and 3.7 mg/mL NPs solution.

The loading concentration of curcumin for the MPs was chosen so that, if all curcumin were to elute at one time point, the concentration of curcumin in the culture media would remain below the cytotoxic concentration [31,43].

The overall sizes of these MPs were measured using ECHO microscope imaging software (ECHO Revolve). Incorporation of curcumin and/or NPs in the MP did not seem to have a significant effect on the size distribution of the microparticles (Figure 2C,D). All MP types had a right-skewed distribution with a peak at approximately 80~100 μm.

### 3.2. Curcumin Release Profile

**Figure 3:**
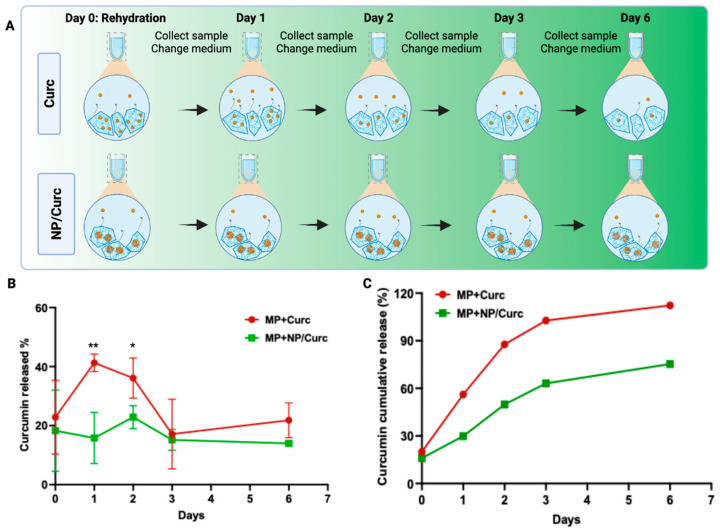
Release profile of curcumin from MPs. (**A**) Scheme of sample collection at different time points. To mimic a sink condition, all solutions were collected and refreshed at each time point. (**B**) The percentage of curcumin released into PBS solution over the course of six days. Data shown are mean ± SD *n* = 4 * *p* < 0.05, ** *p* < 0.01. (**C**) Theoretical cumulative release profile of curcumin. The percentage of release at each time was normalized to the starting mass of curcumin. The cumulative release at each time point was the sum of the time point and all time points preceding it.

To study the release of curcumin from different MP configurations, a sink condition was used to collect all solutions at each time point and replace them with the same volume of fresh solution. As expected, the release of curcumin from GelMA MP containing free curcumin (direct loading) showed a burst release (about 56%) on Day 1 (Figure 3B and Supplemental Materials). By Day 3, about 90% of curcumin had been released from these MPs. In contrast, curcumin loaded into Gelatin NP (NP/Curc) showed reduced release on Day 1 (about 30%). The release showed a sustained fashion up to 75% on Day 6. Fitting the release data to the Korsmeyer-Peppas kinetics model yielded the following results: For MP + Curc, the parameters were K = 0.7183 and *n* = 0.3632 (R^2^ = 0.9579). For MP + NP/Curc, the corresponding values were K = 0.4548 and *n* = 0.3134 (R^2^ = 0.9368). Since *n* < 0.45 for both MP types, the mechanism of curcumin release is governed by Fickian diffusion [44]. Usually, it takes about 5 days for hMSCs to robustly grow on MPs, when the maximal cellular benefits of hMSCs may be harnessed. Given this time frame, only MPs containing NP/Curc have the potential to deliver cells and slowly release curcumin when applied to the wound environment.

### 3.3. Viability of Cells on Microparticle Carriers

**Figure 4:**
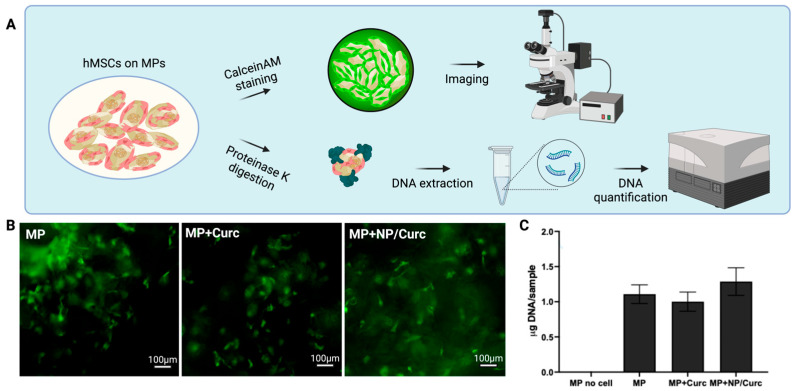
Viability of hMSCs on MP scaffolds. (**A**) Schematic illustration of the assessment of the viability of hMSCs on MPs. (**B**) Calcein AM staining of hMSCs after 6 days of culturing. Scale bar = 100 μm. (**C**) DNA quantification of hMSCs cultured on MPs. Data are shown as mean ± SD (*n* = 4).

The goal of this delivery system is to deliver curcumin and hMSCs simultaneously to the chronic wound environment. To fulfill this aim, these MP configurations must support the viability and growth of hMSCs. To evaluate if the loading of curcumin affected the cell-carrying ability of MPs, hMSCs were seeded on MPs and cultured for 6 days. The viability of hMSCs was detected by a live stain, CalceinAM (Figure 4B). The positive staining (green fluorescence) was almost indistinguishable among the testing groups. To quantify the number of cells on different MPs, MPs cultured with cells on day 6 were digested with proteinase K in the presence of SDS to release DNA. The DNA was extracted, purified, and quantified using the AccuClear dsDNA quantification kit (Figure 4C and Supplemental Materials).

DNA quantification showed no significant differences in the amount of total DNA among different cell carriers MPs. A slight increase in total DNA on MPs containing NP/Curc suggested that lower release of curcumin over culturing time may have a slight advantage for the cells cultured on their surfaces compared with cells on MPs containing free curcumin. Overall, this observation suggests that the inclusion of curcumin to MPs did not compromise the cell-carrying ability of MPs.

### 3.4. Anti-Inflammatory Effects of hMSCs and Curcumin Carried by GelMA Microparticles

**Figure 5:**
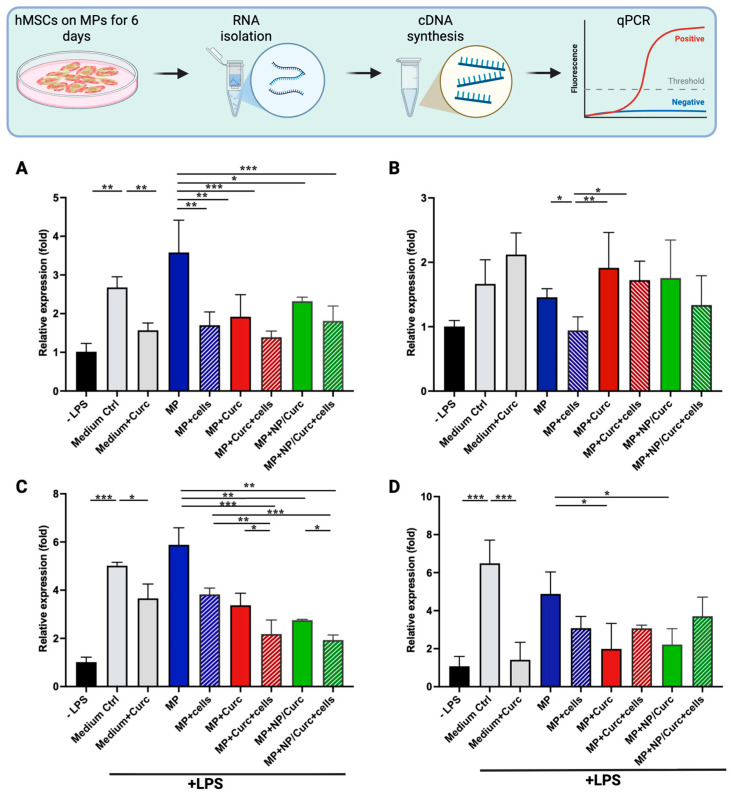
The relative gene expression of pro-inflammatory markers in THP-1 cells in response to LPS stimulation. Differentiated THP-1 cells were treated with LPS (+LPS) in the presence of conditioned medium from MPs (MP), from MPs carrying hMSCs (MP + cells), from MPs loaded with curcumin (MP + Curc), from MPs loaded with curcumin and covered with hMSCs (MP + Curc + cells), from MPs loaded with NPs containing curcumin (MP+/NP/Curc), or from MPs loaded with NP/Curc and covered with hMSCs (MP + NP/Curc + cells) for 24 h. As comparisons, differentiated THP-1 cells were induced by LPS in the presence of medium alone (Medium Ctrl) or medium containing 15 μM curcumin (Medium + Curc). The relative expression of TNFα (**A**), IL-1β (**B**), IL-8 (**C**), or IL-6 (**D**) in THP-1 cells treated with LPS was normalized to differentiated cells without LPS induction (-LPS) by quantitative PCR. Data shown are mean ± SD (*n* ≥ 3) * *p* < 0.05, ** *p* < 0.01, *** *p* < 0.005.

hMSCs are known to modulate inflammation through the secretion of growth factors and cytokines [45]. Separately, curcumin has demonstrated anti-inflammatory properties in both in vitro and in vivo studies [21,22]. To evaluate the anti-inflammatory effects of hMSCs and/or curcumin delivered via GelMA microparticles, conditioned media (CM) from various MP configurations were collected after being cultured for 6 days. Differentiated human monocyte THP-1 cells can be activated to transition into pro-inflammatory macrophages by lipopolysaccharide (LPS), a potent inflammatory trigger [46]. The effect of these conditioned media on the expression of pro- and anti-inflammatory markers in activated THP-1 cells was evaluated after 24 h using quantitative PCR (qPCR) (Figure 5 and Supplemental Materials).

LPS induces the polarization of macrophages toward a pro-inflammatory (M1) state, stimulating the expression of pro-inflammatory markers in THP-1 cells. As shown in Figure 5, LPS significantly induced the expression of TNFα, IL-8, and IL-6. The difference in stimulation of IL-1β was less pronounced, possibly due to the fact that the response of IL-1β often peaked at 2–4 h after LPS treatment [47].

The addition of 15 μM of free curcumin to cell culture medium significantly reduced the LPS-stimulated expression of TNFα, IL-8, or IL-6 (comparing “Medium Ctrl” vs. “Medium + Curc” in Figure 5). The conditioned medium from plain MPs “MP” showed a comparable effect on THP-1 cells to that of “Medium Ctrl”, suggesting that the GelMA hydrogel itself did not confer an anti-inflammatory effect. It was notable that the conditioned medium from hMSCs cultured on MPs (MP + cells) significantly reduced the LPS-stimulated expression of TNFα, IL-1β, or IL-8 (comparing “MP” vs. “MP + cells”). These results indicated that both free curcumin and CM from hMSCs showed anti-inflammatory activity. Curcumin loaded to hydrogel microparticles either directly (Curc) or via the hierarchical constructs (NP/Curc) were likely present in conditioned medium and showed inhibitory effect on TNFα, IL-8, and IL-6 (comparing “MP” vs. “MP + Curc” or “MP + NP/Curc”).

It is observed that the conditioned media from co-delivery constructs (curcumin + hMSCs) showed an additive effect on the inhibition of IL-8 expression (comparing “MP + Curc” or “MP + NP/Curc” vs. “MP + Curc + cells” or “MP + NP/Curc + cells”). No significant difference was observed between directly loaded curcumin or NP loaded curcumin, suggesting a similar amount of curcumin was present in the conditioned media on Day 6.

The loading of curcumin to Gelatin NPs supported a slower release of curcumin from MPs over time (Figure 3). On Day 6, while curcumin was completely released from MP + Curc, curcumin would continue to be released from MP + NP/Curc. Therefore, the hierarchical construct is likely to deliver the benefits of stem cells and curcumin simultaneously after transfer to the wound environment.

The anti-inflammatory (M2) markers, CCL18 and IL-10, were also assessed. Interestingly, no expression of these markers was detected in any samples. This finding suggests that while the conditioned media—containing factors from hMSCs and/or curcumin—effectively reduced the expression of pro-inflammatory markers, they did not induce M2 differentiation in the tested THP-1 cells within the time point of this study.

## 4. Discussion

Current treatments for chronic wounds, such as negative pressure wound therapy (NPWT) and biological grafts, have achieved notable successes [48]. However, given the complex nature of chronic wounds and the growing affected population, there is a pressing demand for highly effective, multi-targeting therapies. To counteract the characteristic deficiency of growth factors and persistent inflammation in these wounds, the design principle of this study is the simultaneous delivery of stem cells and anti-inflammatory compounds.

Drawing upon our previous work with human mesenchymal stem cell (hMSC) culture on GelMA microparticles [17] and gelatin nanoparticle-based drug delivery [39], we sought to develop a hierarchical co-delivery system. While Yu et al. introduced the term “hierarchical” to describe nanoparticles encased within a hydrogel [36], we advanced this concept to simultaneously deliver both a drug and stem cells.

The effectiveness of this hierarchical structure was confirmed by evaluating the release kinetics of curcumin. Pre-loading curcumin into gelatin nanoparticles before encapsulation within the microparticles significantly reduced the initial burst release compared with direct curcumin loading (Figure 3). By the final time point (Day 6), microparticles with directly loaded curcumin had released their entire cargo (100%), whereas those containing nanoparticle-loaded curcumin still retained approximately 25% of the initial curcumin load.

For successful transplantation, hMSCs require time to adhere and expand on the microparticle surface. However, this necessity presents a compromise between the optimal cell culture duration and the retention of the curcumin payload. While a longer culture period allows for greater cell expansion, it also results in the loss of curcumin in vitro before the system is transferred to the wound.

Previous studies indicate that cell number and metabolic activity of cells cultured on gelatin microcarriers peak around Day 5 [9]. Therefore, Day 6 was chosen to maximize cell activity for transplantation in this study. The release profile (Figure 3) showed that the MP + Curc scaffold completely exhausted its curcumin by Day 6, meaning little to no compound would be available in the wound environment.

On the other hand, the inclusion of gelatin nanoparticles attenuated the curcumin release, enabling a sustained delivery. As a result, this hierarchical system expands the viable timeframe for cell culture while still guaranteeing the beneficial presence of curcumin when the scaffold reaches the wound. Future studies will explore earlier time points, such as 3–4 days in vitro or in vivo, to further optimize cell culturing time.

The presence of cultured cells on the microparticles could potentially alter the curcumin release profile. Specifically, the cells may form a barrier around the particles, which could delay the compound’s release. To accurately understand the release of curcumin in the cellular environment, attempts were made to quantify curcumin in the conditioned media from hMSCs cultured on various microparticles. However, reliable quantification proved challenging. Due to interference from the cell culture media, the exact quantity of curcumin could not be determined using standard spectrophotometry or conventional HPLC. A specific, reverse-phase HPLC method is currently in development to enable accurate quantification of curcumin in the conditioned media. [49].

It was observed that the hMSC growth on the surface of microparticles (MPs) was not significantly affected by the curcumin loads (Figure 4). This result suggests that the concentration of curcumin released into the culture medium—the compound with which the cells directly interact—remained below the reported cytotoxic threshold for mesenchymal stem cells [43]. Although the gross viability of the hMSCs was not significantly affected by the curcumin loads, this study did not examine whether the presence of curcumin modulated stem cell functionality. Future research should carefully evaluate the direct effect of curcumin on hMSCs, specifically focusing on dose-dependent outcomes.

To evaluate the co-delivery microparticle (MP) functionality, conditioned media from different MP configurations were applied to pro-inflammatory-stimulated THP-1 cells (Figure 5). As anticipated, media from MPs loaded individually with hMSCs or curcumin inhibited the expression of pro-inflammatory cytokines, including TNF-α, IL-8, and IL-6. More notably, conditioned media from MPs co-loaded with both hMSCs and curcumin exhibited an additive inhibitory effect on IL-8 expression. This enhanced effect likely stems from the distinct yet complementary anti-inflammatory mechanisms of the stem cells and curcumin. hMSCs suppress inflammation indirectly by releasing immunomodulatory factors like IL-10 and Transforming Growth Factor-beta (TGF-β), which can polarize macrophages towards an M2 (anti-inflammatory) phenotype [50]. Curcumin acts more directly by binding to the receptor of the NF-κB signaling pathway in immune cells, including macrophages, to repress the expression of pro-inflammatory cytokines. These different, but potentially overlapping, pathways appear to produce enhanced anti-inflammatory effects compared to either treatment alone. Given that curcumin may directly influence stem cell function [37], further investigation into the interaction between hMSCs and curcumin within this specific MP co-loading construct and how it impacts their combined anti-inflammatory capacity would be a valuable next step.

The absence of an additive effect on TNF-α and IL-6 expression may be due to the single 24-h time point captured in this study. Given that these cytokines peak at different times following LPS activation [51], a more comprehensive investigation of the gene expression profiles and protein levels of various cytokines at different time points is warranted.

Our results provide the initial proof-of-concept for the hierarchical co-delivery system. To realize the therapeutic potential of this platform, its efficacy must be evaluated in a preclinical rodent model of chronic/diabetic wounds [29]. While actively pursuing collaborations for this essential in vivo study, two key areas should be focused on: (1) Process scale-up: scaling up our microparticle fabrication process from the milligram to the gram scale. The synthesis of both the nanoparticles and microparticles can be readily scaled up. (2) Delivery efficiency evaluation: before proceeding to in vivo studies, the impact of larger-scale constructs on the efficiency of drug and cell co-delivery needs to be investigated.

Translating this technology into clinical development requires addressing several critical factors beyond preclinical success. The regulatory landscape for cell-based therapies is still evolving, exemplified by the wide scope of current research. Based on a recent search on ClinicalTrials.gov (search words: stem cell-based therapies and chronic wounds), about 40 active clinical trials are exploring cell-based therapies for chronic wound healing, targeting diverse subjects from diabetic foot ulcers to epithelial repair in chronic obstructive pulmonary disease patients. This variety emphasizes the ongoing need for new and effective therapies. Many clinical case studies utilizing autologous mesenchymal stem cells (MSCs) for chronic wound treatment have shown positive outcomes [52]. If proven effective in preclinical models, our current technology offers a potential advanced delivery platform for autologous stem cells combined with wound-healing compounds. Given its high biocompatibility and moldability, it is envisionable that this system could be used in combination with existing biological extracellular matrix wound dressings, enabling the application of multifaceted therapies to complex chronic wounds.

## 5. Conclusions

In this study, a novel hierarchical delivery system for the anti-inflammatory compound, curcumin, has been developed. Curcumin was first loaded onto gelatin nanoparticles, which were then incorporated into a microparticle scaffold. This hierarchical configuration successfully prevented the burst release of curcumin and ensured its prolonged delivery. Crucially, the microparticle scaffolds also maintained an uncompromised ability to carry and deliver human stem cells, allowing the simultaneous delivery of both stem cells and curcumin. Our in vitro findings successfully demonstrate the feasibility of utilizing this platform to introduce multiple wound healing therapies—essential growth factors and anti-inflammatory compounds—directly into the chronic wound environment.

## Figures and Tables

**Table 1:** QuantiTect primer sets used in this study are listed.

Markers	Genes	Function	Catalog Number of Primers
Glyceraldehyde-3-Phosphate Dehydrogenase	*GAPDH*	Housekeeping gene as an internal control	Hs_GAPDH_2_SG QT01192646
Tumor Necrosis Factor alpha	*TNF*	Pro-inflammation	Hs_TNF_3_SG QT01079561
Interleukin-1 beta	*IL1B*	Pro-inflammation	Hs_IL1B_1_SG QT00021385
Interleukin-6	*IL6*	Pro-inflammation	Hs_IL6_1_SG QT00083720
C-X-C Motif Chemokine Ligand 8 (Interleukin-8)	*CXCL8*	Pro-inflammation	Hs_CXCL8_1_SG QT00000322
C-C motif chemokine ligand 18	*CCL18*	Anti-inflammation	Hs_CCL18_1_SG QT00024066
Glyceraldehyde-3-Phosphate Dehydrogenase	*GAPDH*	Housekeeping gene as an internal control	Hs_GAPDH_2_SG QT01192646

## Data Availability

Raw data have been submitted as Supplemental Materials.

## Supplementary Materials

The following supporting information can be downloaded at: https://www.mdpi.com/article/10.3390/pharmaceutics17121549/s1. The raw data of Figure 3, Figure 4 and Figure 5 are provided as Supplemental Materials.
